# Turbulent blood dynamics in the left heart in the presence of mitral regurgitation: a computational study based on multi-series cine-MRI

**DOI:** 10.1007/s10237-023-01735-0

**Published:** 2023-07-03

**Authors:** Lorenzo Bennati, Vincenzo Giambruno, Francesca Renzi, Venanzio Di Nicola, Caterina Maffeis, Giovanni Puppini, Giovanni Battista Luciani, Christian Vergara

**Affiliations:** 1 Department of Surgery, Dentistry, Pediatrics, and Obstetrics/Gynecology, University of Verona, Piazzale Ludovico Antonio Scuro 10, 37134 Verona, Italy; 2 Division of Cardiac Surgery, Department of Surgery, Dentistry, Pediatrics, and Obstetrics/Gynecology, University of Verona, Piazzale Stefani 1, 37126 Verona, Italy; 3 Department of Radiology, University of Verona, Piazzale Stefani 1, 37126 Verona, Italy; 4 LaBS, Dipartimento di Chimica, Materiali e Ingegneria Chimica “Giulio Natta”, Politecnico di Milano, Piazza Leonardo da Vinci 32, 20133 Milan, Italy

**Keywords:** Turbulence, Hemolysis, Blood washout, Mitral regurgitation, Computational fluid dynamics, Multi-series cine-MRI

## Abstract

In this work, we performed a computational image-based study of blood dynamics in the whole left heart, both in a healthy subject and in a patient with mitral valve regurgitation. We elaborated multi-series cine-MRI with the aim of reconstructing the geometry and the corresponding motion of left ventricle, left atrium, mitral and aortic valves, and aortic root of the subjects. This allowed us to prescribe such motion to computational blood dynamics simulations where, for the first time, the whole left heart motion of the subject is considered, allowing us to obtain reliable subject-specific information. The final aim is to investigate and compare between the subjects the occurrence of turbulence and the risk of hemolysis and of thrombi formation. In particular, we modeled blood with the Navier–Stokes equations in the arbitrary Lagrangian–Eulerian framework, with a large eddy simulation model to describe the transition to turbulence and a resistive method to manage the valve dynamics, and we used a finite element discretization implemented in an in-house code for the numerical solution.

## Introduction

The pathologies affecting the left heart (LH) are the most common cause of death in the world Tsao et al. (2022). One of these is mitral valve regurgitation (MVR), a condition leading to a formation of a regurgitant jet in the left atrium during the systolic phase due to an incomplete closure of the mitral valve leaflets. The formation and the development of the regurgitant jet may give rise to: i) the presence of highly disturbed or even turbulent atrial flow that can lead to hemolysis in the atrium (Dyverfeldt et al. 2011; Sugiura et al. 2018) and ii) washing out of stagnant blood in the atrium that could prevent thrombi formation (Cresti et al. 2019).

These phenomena are difficult to describe and quantify in the clinical practice. On the one hand, although clinical measures such as the *regurgitant volume* and the *regurgitant fraction* may provide significant information about the global cardiac function; they are not able to capture local features such as 3D velocity distribution and wall shear stresses (Kon et al. 2004; Myerson et al. 2016). Moreover, the space and time resolution of the available imaging techniques, such as four-dimensional flow magnetic resonance imaging (MRI) or phase-contrast MRI, is not nowadays enough accurate to capture small-scale features as recirculation areas, regions of transition to turbulence and small coherent structures (Ngo et al. 2019).

In this respect, computational methods can noninvasively provide quantitative information about the local pressure gradients, the velocity patterns and the shear forces, contributing to a better understanding of the cardiovascular system (Augst et al. 2007; Groen et al. 2007; Bazilevs et al. 2009; Esmaily et al. 2012; Schrauwen et al. 2016; Liu et al. 2016; Colebank et al. 2021). In particular, computational models applied to LH have contributed to a better knowledge of the cardiac physiopathology (Ma et al. 2013; Caballero et al. 2018; Fuchsberger et al. 2019; Feng et al. 2019; Karabelas et al. 2022; Meschini et al. 2019, 2021; Viola et al. 2022; Bucelli et al. 2023) and to model and predict the outcomes of valve prostheses or surgical interventions (Spühler et al. 2018; Luraghi et al. 2019; Caballero et al. 2020; Gallo et al. 2022). Such methods can be broadly grouped in two categories: *fluid structure interaction* (FSI) models (Meschini et al. 2019; Caballero et al. 2018; Viola et al. 2022; Bucelli et al. 2023) and *Computational Fluid Dynamics (CFD) with prescribed wall motion*. For the latter, the prescribed motion could be obtained either from an electromechanical simulation (Augustin et al. 2016; Karabelas et al. 2018; This et al. 2020; Zingaro et al. 2022, 2023) or by dedicated time-resolved medical images (*Dynamic Image-based CFD*, DIB-CFD). The latter approach has become, in the last decade, a valid alternative to FSI models when sufficiently detailed dynamic medical images are available (Seo et al. 2014; Su et al. 2016; Chnafa et al. 2014; Bavo et al. 2016, 2016; Chnafa et al. 2016; Fumagalli et al. 2020, 2022). In particular, regarding DIB-CFD studies investigating MVR, we mention works where the authors tested and compared different types of mitral valve prolapse (Collia et al. 2019), different degrees of MVR (Obermeier et al. 2022), different functional changes of the ventricle and atrium in response to MVR (Bennati et al. 2023) and different effects of MVR in the right heart (Bonini et al. 2022). However, none of these studies was performed on a fully patient-specific LH geometry and displacement (ventricle + atrium + mitral valve + aortic valve + aortic root). Moreover, none of them investigated the transition to turbulence or the risk of hemolysis and the prevention from thrombi formation in the atrium.

In this context, the present study has two principal aims:

(i) to perform a DIB-CFD simulation of the whole heartbeat on a healthy subject and on a patient with MVR;
(ii) to investigate the transition to turbulence, the risk of hemolysis formation and the prevention from thrombi formation with respect to the healthy subject.

The main improvements and novelties of this work are:

(i) a fully LH patient-specific DIB-CFD simulation with imposed motion of ventricle, atrium and aortic root together with the geometries of the mitral and aortic valve, all reconstructed from multi-series (i.e., Long Axis, Short Axis and mitral valve Rotated series) cine-MRI during the whole heartbeat. In particular, unlike the works of Chnafa et al. (2014, 2016), we included both the aortic and mitral valve geometries;
(ii) the investigation of the transition to turbulence in the MVR case, so far investigated only in physiological cases (Chnafa et al. 2014, 2016);
(iii) the study of the risk of hemolysis in the atrium and the prevention from thrombi formation with respect to the healthy subject. These phenomena, so far, has not been investigated in the presence of native valve with MVR by means of a computational study.

Moreover, the novelty with respect our previous work (Bennati et al. 2023) is the simulation of the whole heartbeat.

The significance of our results has been supported by the validation with echo color Doppler (ECD) measures in the healthy subject and by a qualitative comparison with a cine-MRI flow pattern in the MVR case.

## Methods

In this section, we first described the multi-series cine-MRI at disposal and the acquisitions of the ECD measurements; after that, we detailed the reconstruction techniques used to obtain the patient-specific geometries and displacements of the left ventricle (LV), left atrium (LA), aortic root (AR), mitral valve (MV) and aortic valve (AV); then, we briefly reported the mathematical and numerical methods used in this work; finally, we introduced the quantities of interest that has been analyzed in the Results section.

### Available cine-MRI and ECD acquisitions

Cardiac multi-series cine-MRI data of two subjects were provided by the Department of Radiology of Borgo Trento Hospital, Verona, Italy. Ethical review board approval and informed consent were obtained from all subjects. In particular, we acquired dynamic images, consisting of 30 acquisitions per heartbeat, of a healthy subject (H) and of a patient with a severe MVR due to a posterior leaflet prolapse (R). In Table 1, we reported some information about the two subjects, including the heart rate^1^, height, weight and body surface area (BSA) (Du Bois and Du Bois 1989).

**Table 1:** For each patient, we reported the values of heart rate, height, weight and BSA

Subject	Heart rate [*BPM*]	Height [*m*]	Weight [*kg*]	BSA [$m^2$]
H	66	1.93	84	2.14
R	75	1.84	105	2.28

**Fig. 1:**
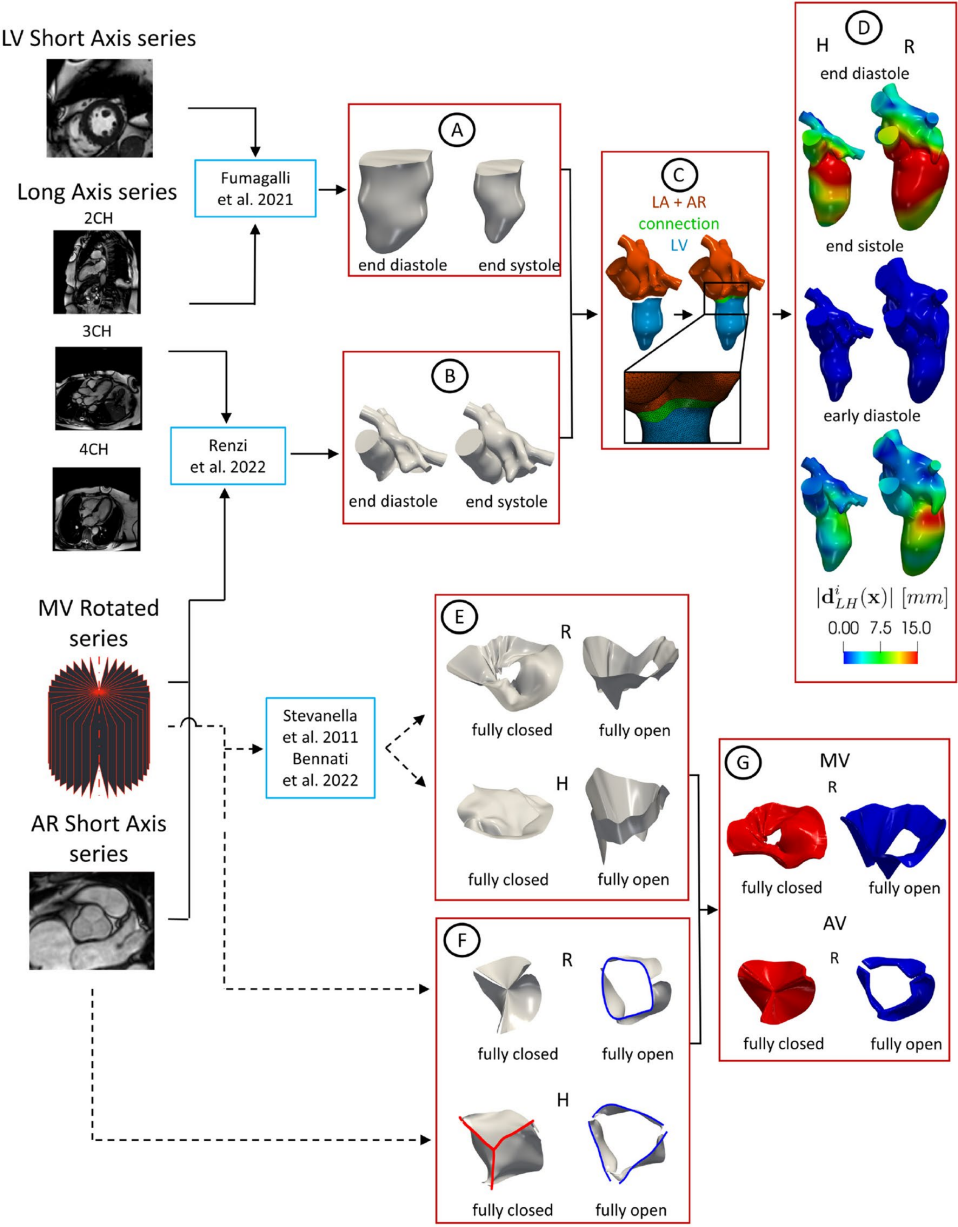
Flowchart to reconstruct the geometries of LH and valves and their displacements. Continuous lines refer to the steps followed to reconstruct the LH walls and the dashed lines to the reconstruction of the valves. A: Geometric reconstruction of the LV endocardium for all the 30 frames by adopting the strategy described in Fumagalli et al. (2022) (here we reported the geometries at two representative frames as an example). B: Geometric reconstruction of AR and LA for all the 30 frames by adopting the strategy proposed in Renzi et al. (2022) (two representative frames reported as an example). C: Creation of a connection to merge LV with AR and LA to obtain the final LH geometry. D: Registration of the displacement field $\textbf{d}^i_{LH}(\textbf{x})$ with respect to the end systolic configuration (magnitude of $\textbf{d}^i_{LH}(\textbf{x})$ reported at three representative frames as an example). E: Geometric reconstruction of MV in the fully open and closed configurations by adopting the method proposed in Stevanella et al. (2011). F: Geometries of AV in the fully open and closed configurations obtained by adaptation of zygote geometry to the annulus segmented from the cine-MRI in the closed (red) and open (blue) frames, respectively. Notice that the annulus of the closed AV of patient R was obtained through algorithms based on the closest point distances (Fedele and Quarteroni 2021). G: Valve configurations after the extrusion needed for the resistive numerical method (we reported as representative case only patient R)

The acquisitions were performed with the Achieva 1.5T (TX) - DS (Philips, Amsterdam, the Netherlands) technology. Specifically, for each patient, we have at disposal different multi-series images: the *Short Axis* series of LV, the *Long Axis* series (2CH, 3CH and 4CH views) and the *Rotated* series of MV. Moreover, for subject H we have at disposal also the Short Axis series of AR. Each series has a time resolution of 30 frames/cardiac cycle. Below we reported the other specific characteristics of each series (see Fig. 1, left):

- *LV Short Axis*: volumetric series made of 15 slices; thickness and distancing of 8 mm along the LV main axis; spatial resolution of 1 mm;
- *Long Axis*: set of single slices, two-dimensional acquisitions on the two-chamber (2CH), three-chamber (3CH) and four-chamber (4CH) planes; space resolution of 1 mm; slice thickness of 8 mm;
- *Mitral valve*: two-dimensional series of 18 evenly rotated (one every 10 degrees) planes around the axis passing through the annular center and aligned with the left ventricle apex; spatial resolution of 1.25 mm; slice thickness of 8 mm;
- *AR Short Axis*: volumetric series made of 4 slices; thickness and distancing of 8 mm along the aortic root main axis; spatial resolution of 1 mm.

After the image acquisitions, patient R underwent to a surgical operation to restore a correct heart function.

We point out that the Long Axis series and the Short Axis series of LV are *standard* cine-MRI data that are routinely acquired in the clinical procedure. Instead, the Short Axis series of AR and the Rotated series of MV represent *ad hoc* advanced cine-MRI acquisitions.

**Fig. 2:**
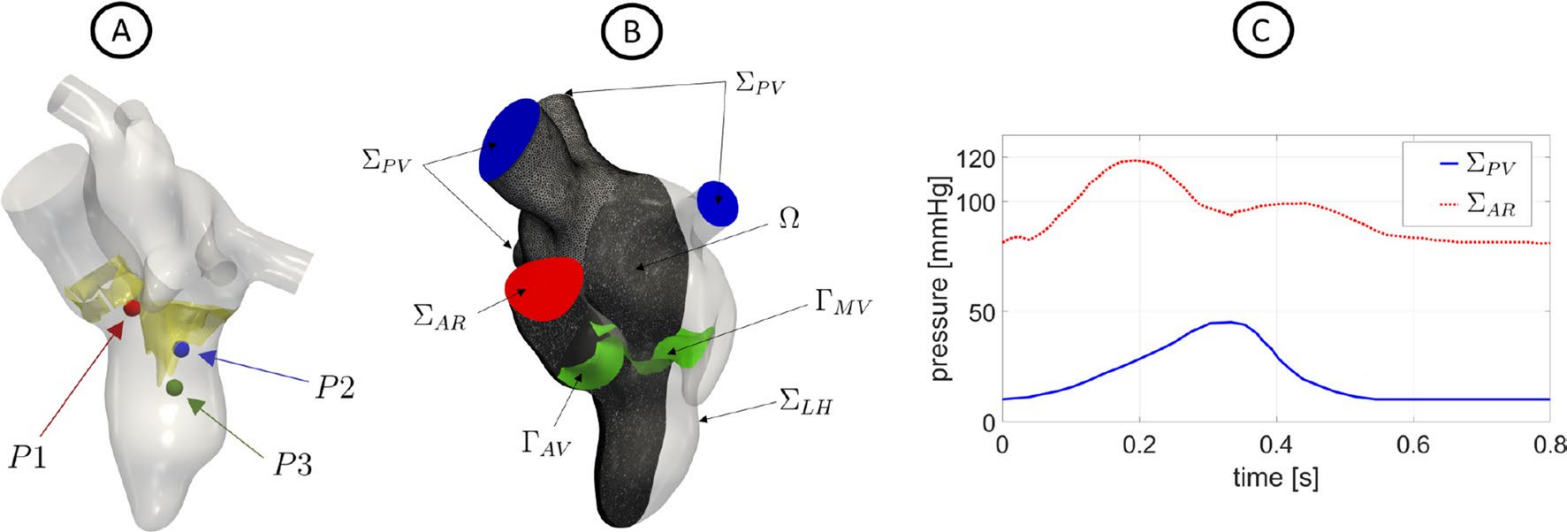
A: Location of the points where ECD measures were acquired. B: Computational domain $\Omega$ with its boundaries. In green, we reported the aortic and mitral valves $\Gamma _{AV}$ and $\Gamma _{MV}$. The computational mesh of patient R is also displayed. C: Trend in time of the pressures imposed at $\Sigma _{PV}$ (for scenario R) and $\Sigma _{AR}$ (for H, the curve at the AR outlet has been suitably adapted based on its heartbeat)

After the cine-MRI acquisitions, ECD measurements with a EPIQ CVx ultrasounds scanner and linear 8MHz probe (Philips Ultrasound, Bothell, WA) were taken from subject H . The velocity measures were acquired at the peak instants at three different locations: *P*1: $1\ cm$ upstream the AV base; *P*2: $1.6\ cm$ downstream the MV annulus; and *P*3: $5.5\ cm$ far from the LV apex, see Fig. 2A.

### Geometric reconstruction of the left heart internal wall surfaces

In this section, we describe a novel framework to reconstruct the LH geometry and displacement. This is based on combining two different reconstruction techniques proposed so far for LV and for LA/AR, respectively. The entire procedure is presented in Fig. 1, from step A to step C.

Regarding the LV reconstruction, we adopted the strategy described in Fumagalli et al. (2022). Starting from the Short Axis series of LV, we merged them with the Long Axis acquisitions (2CH, 3CH and 4CH views) to obtain new enhanced time-dependent series of volumetric images with a uniform space resolution of $1\ mm$ in all directions. From these enhanced images, we segmented and reconstructed the shape of the LV endocardium in all the 30 frames by using the algorithm proposed in Fetzer et al. (2014) and implemented in the *Medical Image Toolkit* (MITK) open-source software (www.mitk.org), see step A in Fig. 1.

After, we reconstructed the shape of AR and LA for all the 30 frames by using a cine-MRI multi-image-based reconstruction algorithm, proposed in Renzi et al. (2022) for the right heart and implemented in the *Vascular Modeling Toolkit* (VMTK) (www.vmtk.org) (Antiga et al. 2008; Fedele and Quarteroni 2021). This is based on manually tracing the contours of AR and LA from the MV Rotated, Long Axis and, when available, AR Short Axis series. For each frame, a 3D point cloud was obtained that was turned into a surface mesh of triangles, see step B in Fig. 1.

Then, for each reconstructed frame, we avoided possible geometrical mismatches by leaving a small gap between LV and AR + LA during their reconstruction, see step C in Fig. 1. In order to merge them, we created a connection in VMTK Fedele and Quarteroni (2021) to obtain the complete geometry of LH (see Fig. 1, step C) that was after remeshed and smoothed in *MeshMixer* (https://www.meshmixer.com). Also notice that the 3D system of coordinates is absolute for all the series since the patients were kept supine throughout the examination. Furthermore, all the series have been acquired by the same machine within a few minutes of each other. Moreover, we assumed that no temporal mismatches were present between the series, since the maximum discrepancy of the frequency with respect to the short axis series of the left ventricle (used to evaluate the heartbeat of the patients, reported in Table 1) was below 2%.

Subsequently, we registered the displacement of each frame with respect to the end systolic configuration by exploiting the non-affine B-splines algorithm implemented in the *Elastix* open-source library (http://elastix.isi.uu.nl) Klein et al. (2010) used and validated in Bennati et al. (2023). The outputs are the surface mesh of LH at the end systolic instant together with the displacement fields $\textbf{d}^i_{LH}(\textbf{x}),\,i=1,\ldots ,30,$ computed for all the 30 frames with respect to such systolic configuration. In Fig. 1, step D, we reported the magnitude of $\textbf{d}^i_{LH}(\textbf{x})$ at three representative frames.

### Geometric reconstruction of the valves

In this section, we describe the geometric reconstruction of the valves. The entire procedure is reported in Fig. 1, steps D and E. Regarding the MV reconstruction, starting from the Rotated series of MV, we reconstructed its shape in the fully closed (FC) and fully open (FO) configurations by using the method proposed in Stevanella et al. (2011) (see step E in Fig. 1), where the authors performed a structural analysis, see also (Gaidulis et al. 2019). This method is based on tracing the valve leaflets in each plane to obtain a 3D point cloud that was after fitted with a B-spline and then turned into a surface mesh of triangles in *MATLAB* (www.mathworks.com), see (Bennati et al. 2023) for details, where this MV reconstruction procedure has been, for the first time, applied to DIB-CFD simulations.

Concerning AV, the cine-MRI at disposal did not allow a complete reconstruction of the patient-specific leaflets. For such reason, what we have done is to reconstruct the patient-specific annulus of H and R in the open configuration and to adapt the aortic valve geometry taken from *Zygote solid 3D heart model* (a complete geometry reconstructed from CT scans representing an average healthy heart, https://www.zygote.com) to our reconstructions. In particular, the FO configurations were geometrically deformed in order to match the annulus with that segmented from our cine-MRI (from the AR Short Axis series for subject H and from the Rotated series of MV for patient R), see step F in Fig. 1. The AV FC configuration was obtained for subject H by means of the same procedure used for FO and for patient R by exploiting algorithms based on the closest point distances (Fedele and Quarteroni 2021), see step F in Fig. 1.

After, to guarantee a perfect adhesion between the valves and the reconstructed LH geometry at the end systolic instant (see step D in Fig. 1), we placed the valves in the corresponding valvular planes of the LH geometry and we calculated the minimum distance of the valves annulus with respect to the walls. Then, we harmonically extended this distance over all the valve surfaces by using VMTK (Fedele and Quarteroni 2021). Finally, we warped the valves according to such displacements.

As final step of our preprocessing flowchart, panel G in Fig. 1 shows how we extruded the leaflet surfaces in order to provide the valve thickness needed by the numerical method, see Sect. 2.4. This step was in practice performed within the finite element solver, see Sect. 2.4.

### Mathematical and numerical modeling

Blood was modeled as an incompressible, homogeneous, Newtonian fluid with density $\rho = 1.06\cdot 10^3\ kg/m^{3}$ and dynamic viscosity $\mu = 3.5\cdot 10^{-3}\ Pa\cdot s$, described by the Navier–Stokes (NS) equations, see (Quarteroni et al. 2007; Quarteroni 2013). To solve NS in the moving LV and LA we used the arbitrary Lagrangian–Eulerian (ALE) framework (Donea et al. 1982), whereas the presence of the valves was accounted for by using the resistive immersed implicit surface (RIIS) method (Fernández et al. 2008; Fedele et al. 2017). To evaluate the transition to turbulence, we employed the $\sigma$-LES method proposed for ventricular blood dynamics in Nicoud et al. (2011) and successfully used in different hemodynamic applications (Lancellotti et al. 2017; Vergara et al. 2017; Stella et al. 2019).

The displacement of LH $\textbf{d}_{LH}^i(\textbf{x})$ is derived in time and used to compute the wall velocity to prescribe as boundary condition for the NS equations. Since $\textbf{d}_{LH}^i(\textbf{x})$ has been obtained only at the 30 MRI acquisition times , we employed a spline interpolation to obtain $\textbf{d}_{LH}(\textbf{x},t)$ for all *t* $\in$ [0, *T*] where *T* is the duration of the heartbeat, equal to $0.9\ s$ for subject H and $0.8\ s$ for patient R, see Table 1. According to the ALE framework, at each time, the fluid domain $\Omega (t)$ is obtained by extending $\textbf{d}_{LH}(\textbf{x},t)$ into $\Omega$ through the solution of a linear elastic problem (Stein et al. 2003). See (Bennati et al. 2023) for further details.

The entire domain with its boundaries is displayed in Fig. 2B. In particular, $\Sigma _{LH}$ represents the internal wall surfaces of LH, $\Sigma _{AR}$ and $\Sigma _{PV}$, the outlet and inlet sections of the aortic root and pulmonary veins, respectively. In green, instead, we reported the surfaces of the aortic ($\Gamma _{AV}$) and mitral ($\Gamma _{MV}$) valves.

Thus, the ALE NS equations in the known domain $\Omega (t)$ are solved to find the pressure *p* and the blood velocity $\textbf{u}$:

1 $$\begin{aligned} {\left\{ \begin{array}{ll} \rho \dfrac{\partial \textbf{u}}{\partial t} + \rho \left( \textbf{u} - \textbf{u}_{ALE}\right) \cdot \nabla \textbf{u} \ + \\ \quad - \left( \mu +\mu _{sgs}\right) \Delta \textbf{u} + \nabla p \ + \\ \quad +\ \sum _{i=AV,MV} \dfrac{R_{\Gamma }}{\varepsilon _{\Gamma }}\left( \textbf{u}-{\textbf{u}_{ALE}}\right) \delta _{\Gamma _i} = \textbf{0}\, \ {} &{} \text {in}\ \Omega (t), \\ \nabla \cdot \textbf{u} = 0 \ {} &{} \text {in}\ \Omega (t),\\ \textbf{u}=\dfrac{\partial \textbf{d}_{LH}}{\partial t} &{} \text {on}\ \Sigma _{LH}(t),\\ \end{array}\right. } \end{aligned}$$

with a null initial condition in $\Omega (0)$. $\mu _{sgs}$ is the subgrid viscosity of the $\sigma$-model (Nicoud et al. 2011), whereas $\delta _{\Gamma _i}$ is a smoothed Dirac delta function representing a layer, with thickness $2\varepsilon _{\Gamma }$, around the surface of the valve $\Gamma _i,\,i=AV,MV$ (Fedele et al. 2017; Fumagalli et al. 2020) and $R_{\Gamma }$ is a resistance coefficient. In our numerical experiments, we set $R_{\Gamma }=10^5$ $kg/m\cdot s$ and $\varepsilon _{\Gamma }=0.75\ mm$ (Bennati et al. 2023; Bucelli et al. 2023).

The valve dynamics has been modeled in an on–off modality, where the reconstructed leaflets opened and closed instantaneously according to the following rule (Quarteroni et al. 2017):

- if $\Delta P > 0\rightarrow$ valve opens,
- if $Q_{AV} < 0\rightarrow$ AV closes,
- if $Q_{MV} < 0\ \& \ t > 0.77\ s \rightarrow$ MV closes

where $\Delta P$ is the difference between upstream and downstream pressures and $Q_{AV}$ and $Q_{MV}$ are the flow rates through AV and MV, respectively. Notice that we needed to add the condition $t > 0.77\ s$ for the MV closure since the condition $Q_{MV} < 0$ alone would be satisfied also before the real MV closure, due to blood flow reversal through the mitral valve during the ventricular diastole. Such flow reversals were reported also in other computational studies (Caballero et al. 2018; Karabelas et al. 2022; Broomé et al. 2013). Since we are not modeling the closure dynamics, we need to control the closure with a check on the flow rate ($Q_{MV} < 0$), we needed to add this further constraint in order to close the valve at the right instant. In particular, in our work the last flow reversal occurred at $0.77\ s$^2^.

Moreover, in order to guarantee a perfect adhesion between the valves and LH internal wall surfaces, we imposed that both valves move in accordance with the ALE movement of LH.

Regarding the remaining boundary conditions of system (1), we prescribed a Neumann condition in the normal direction by imposing: on $\Sigma _{PV}$ a constant pressure of $10\ mmHg$ for H (Wiggers 1923; Brath and Eisenach 2000) and a time-dependent evolution for R (Caballero et al. 2018), see Fig. 2C; a time-dependent physiological pressure (Caballero et al. 2018; Brath and Eisenach 2000) at $\Sigma _{AR}$ for both cases, see Fig. 2C. In the tangential direction, in order to avoid possible backflows instabilities, we prescribed a null velocity (Bertoglio et al. 2018).

To numerically solve system (1), we used first-order finite elements together with first-order semi-implicit discretization in time (Quarteroni et al. 2017). The numerical scheme was stabilized by means of the SUPG/PSPG scheme (Tezduyar and Sathe 2003) implemented in the multiphysics high-performance library $life^x$ (Africa 2022; Africa et al. 2023) (https://lifex.gitlab.io/) based on the deal.II core (Arndt et al. 2021). We run the simulations using 384 parallel processes on the GALILEO100 supercomputer (https://www.hpc.cineca.it/hardware/galileo100) at the CINECA high-performance computing center (Italy).

Tetrahedral meshes were generated in VMTK with an average mesh element size equal to $1.1\ mm$ for H and $1.5\ mm$ for R, with a local refinement of $0.3\ mm$ close to valves, corresponding to about 1.8M degrees of freedom in both the cases (see Fig. 2B for the mesh of R). The time step $\Delta t$ was equal to $5\cdot 10^{-4}\ s$. We performed a mesh convergence test ensuring that no significant differences may be found by using a finer mesh or a smaller time step. Specifically, the values of average mesh element size of H ($1.1\ mm$) and R ($1.5\ mm$) were determined after a mesh refinement study for the investigation of WSS acting in a region in correspondence with LVOT for H and in the atrium for R, at the instant of peak systolic flow rate. In particular, starting from $h=1.1\ mm$ (H) and $h=1.5\ mm$ (R) we checked that the spatial average of WSS (calculated over such regions) did not change (up to a tolerance of 0.5%) when we increased the number of tetrahedra of 10%. After we performed the analysis on the values of $\Delta t$ and we noticed that starting from $\Delta t = 5\cdot 10^{-4}\ s$, halving this value the spatial average of WSS did not change (up to a tolerance of 4%).

To assess the LES quality (and consequently of the average mesh element size), we used the Pope criterion (Pope 2004). In particular, we computed the quantity $M(\textbf{x},t)$:

2 $$\begin{aligned} M(\textbf{x}, t) = \dfrac{k_{sgs}(\textbf{x},t)}{(k_{sgs}(\textbf{x},t) + k_{res}(\textbf{x},t))}, \end{aligned}$$

where $k_{sgs} = \mu _{sgs}(\textbf{x},t)^2 / (C\cdot \Delta \cdot \rho )^2$ (Sagaut and Lee 2002) is the turbulent kinetic energy related to the unresolved scales, where $C = 1.5$ is a LES constant (Nicoud et al. 2011) and $\Delta$ is the average mesh element size; $k_{res}$ is the turbulent kinetic energy of the resolved scales. Values of *M* below the threshold of 20% indicate that the LES is sufficiently resolved (Pope 2004; Chnafa et al. 2014, 2016). In our results, we found that the average in time of LH volumes with M below the threshold was 89% (H) and 87% (R) confirming that, with such values of *h*, we were able to capture more than 80% of the turbulent kinetic energy in at least 87% of the left heart. This result is in accordance with that found in other ventricular LES studies, see, e.g., (Chnafa et al. 2014).

We simulated 13 heartbeats and we discarded the first one to remove the influence of the null initial condition.

### Quantities of interest

To describe and quantify the transition to turbulence, risk of hemolysis and thrombi formation in the two scenarios, we quantitatively analyzed and evaluated the *ensemble* pressure and velocity numerical solutions of (1), i.e., the average calculated over 12 heartbeats. Starting from the ensemble velocity, we also introduce the following ensemble quantities:

- Wall Shear Stresses (WSS) is a function of space and time representing the viscous forces, per unit of area, exerted by the blood on the walls (Katritsis et al. 2007). In particular, we computed the time average wall shear stresses, TAWSS($\textbf{x}$). High values of TAWSS may damage the LH endocardium and trigger possible remodeling processes (Janse 1997); TAWSS is defined as follows:
  $$\begin{aligned} TAWSS(\textbf{x}) = \dfrac{1}{T} \int _{0}^{T}\left\| WSS(\textbf{x},t)\right\| dt \end{aligned}$$
- Relative residence time (RRT) is a function of space providing a surrogate information about the effective residence time spent by particles close to the myocardial wall. High values of this quantity may be a marker of stagnant flow (Riccardello et al. 2018); RRT is defined as follows (Himburg et al. 2004):
  $$\begin{aligned} RRT(\textbf{x}) = \dfrac{1}{(1-2 OSI(\textbf{x}))TAWSS(\textbf{x})} \end{aligned}$$
  where
  $$\begin{aligned} OSI(\textbf{x}) = \dfrac{\left\| \int _{0}^{T}WSS(\textbf{x},t)dt\right\| }{\int _{0}^{T}\left\| WSS(\textbf{x},t)\right\| dt} \end{aligned}$$
- E-wave propagation index (EPI) is the ratio between the space covered by the blood jet developing at the MV orifice during the E-wave and the length of the ventricle at the end diastolic configuration. Values of EPI < 1 could indicate an incomplete apical washout, leading to possible left ventricle thrombus formation (Harfi et al. 2017);
- Standard Deviation (SD) at each time and space of the blood velocity with respect to its ensemble value. This allowed us to quantify and localize the regions characterized by marked transition to turbulence (Vergara et al. 2017; Stella et al. 2019);
- Global Turbulent Kinetic Energy (GTKE, known also as Integrated Fluctuating Kinetic Energy) at each time, quantifying the velocity fluctuations by means of the fluid Reynolds stress tensor (Baldwin et al. 1993; Chnafa et al. 2016);
- Maximum tangential stress $\tau _{max}$ of the fluid Reynolds stress tensor (Baldwin et al. 1993) is a function of space and time quantifying the fluctuating (turbulent) forces exerted among the fluid layers and thus the possible damage caused to blood cells. Notice that values greater than $800\ Pa$ are considered to create the conditions that promote hemolysis (Lu et al. 2001).

## Results

**Fig. 3:**
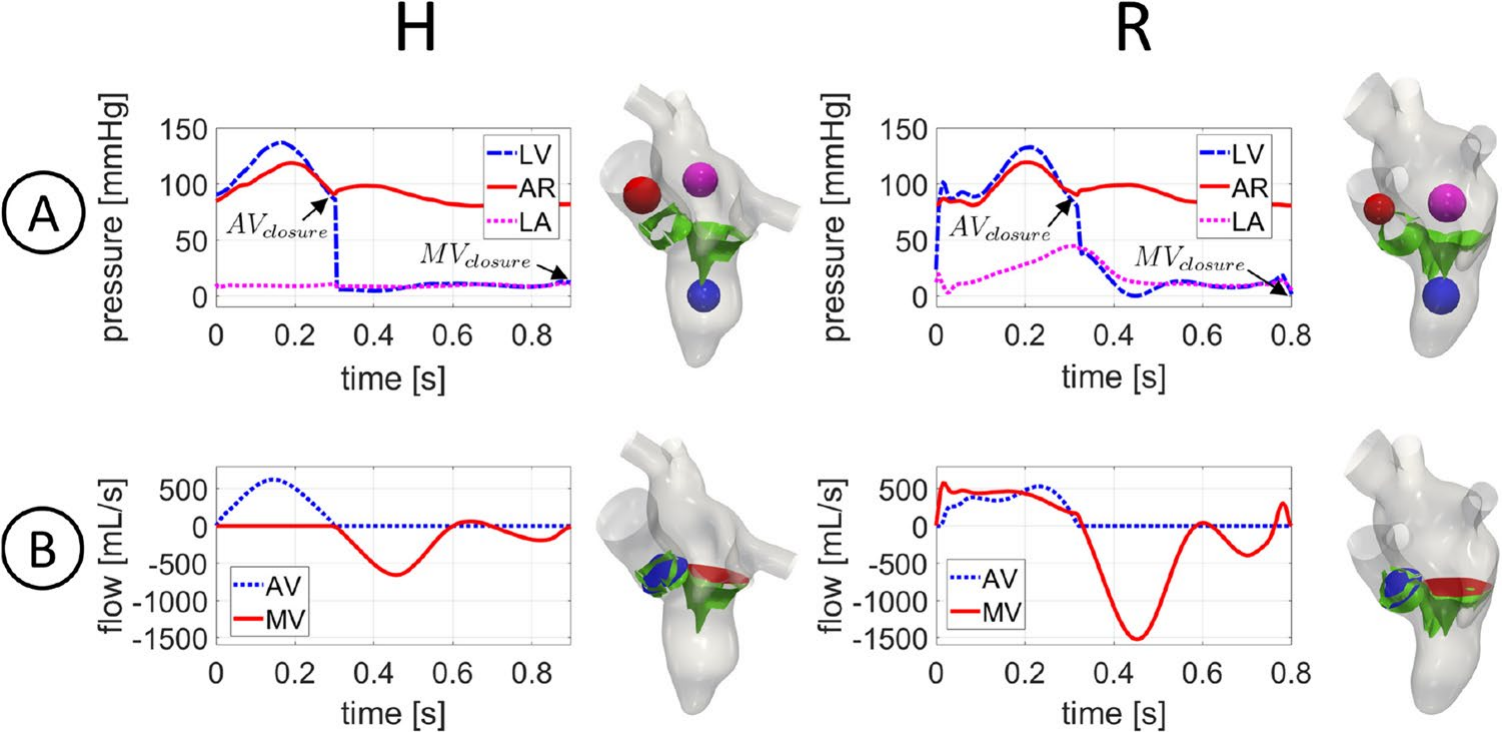
For each scenario: A: trend in time of the mean ensemble ventricle (LV), aortic (AR) and atrial (LA) pressures in the blue, red and magenta spheres, respectively; B: trend in time of the ensemble flow rates through AV (blue section) and MV (red section). $AV_{closure}$: closure of aortic valve; $MV_{closure}$: closure of mitral valve

In Fig. 3A, we reported the trend in time of the mean ensemble ventricular (LV), aortic (AR) and atrial (LA) pressures computed in the blue, red and magenta spheres, respectively.

Notice that the maximum systolic drop $\Delta P_{AV}$ between LV (blue line) and AR (red line) pressures was reached at the middle of systole for H and slightly later (about $60\ ms$) for R, with values of $22\ mmHg$ and $14\ mmHg$, respectively. As expected, R featured a lower $\Delta P_{AV}$ with respect to H, due to the presence of regurgitation (Gaasch and Meyer 2008). The closure of the aortic valve occurred at $0.30\ s$ for H (i.e., at $33\%$ of the heartbeat) and at $0.32\ s$ for R (i.e., at $40\%$ of the heartbeat). During the diastolic phase, the ventricle pressure remained almost constant in H, with a peak of pressure drop $\Delta P_{MV}$ between LA (fucsia line) and LV (blue line) during the E-wave equal to $3.6\ mmHg$ and $12.5\ mmHg$ for H and R, respectively. The larger peak of $\Delta P_{MV}$ featured by R with respect to H was in accordance with (Mokadam et al. 2011). The closure of the mitral valve occurred at $0.9\ s$ and $0.8\ s$ for H and R, respectively. Regarding the atrial pressure, in accordance with the boundary conditions imposed at PVs, we observed in the healthy case a constant value of $10\ mmHg$ during the whole heartbeat, whereas in the regurgitant scenario the pressure increased up to $40\ mmHg$.

In Fig. 3B, we reported the ensemble flow rates evaluated through AV (blue plane) and MV (red plane) for the two scenarios. During the systolic phase, the AV flow rate reached a maximum of $617\ mL/s$ for H and $530\ mL/s$ for R. Notice that the peak of the AV flow rate was reached at the same instant of maximum systolic $\Delta P_{AV}$ in both the scenarios. The flow rate through MV in R featured a peak of $574\ mL/s$. During diastole, in the MV flow rate curve we recognized the E-wave (first minimum), the A-wave (second minimum) and the diastasis (middle stage of diastole). The E-wave featured a maximum flow rate absolute value of $650\ mL/s$ and $1525\ mL/s$ for H and R, respectively. The higher diastolic MV flow rate in R was in accordance with (Schiller et al. 1993). During diastasis, the MV flow rate decelerated until a slightly reversal at $0.63\ s$ (i.e., 70% of the heartbeat) and $0.60\ s$ (i.e., $75\%$ of the heartbeat) for H and R, respectively. During the A-wave, the flow rate reached absolute values of $190\ mL/s$ for H and $392\ mL/s$ for R. After the A-wave, the second flow reversal through MV occurred at $0.77\ s$ (i.e., 96% of the heartbeat) in the regurgitant scenario.

**Fig. 4:**
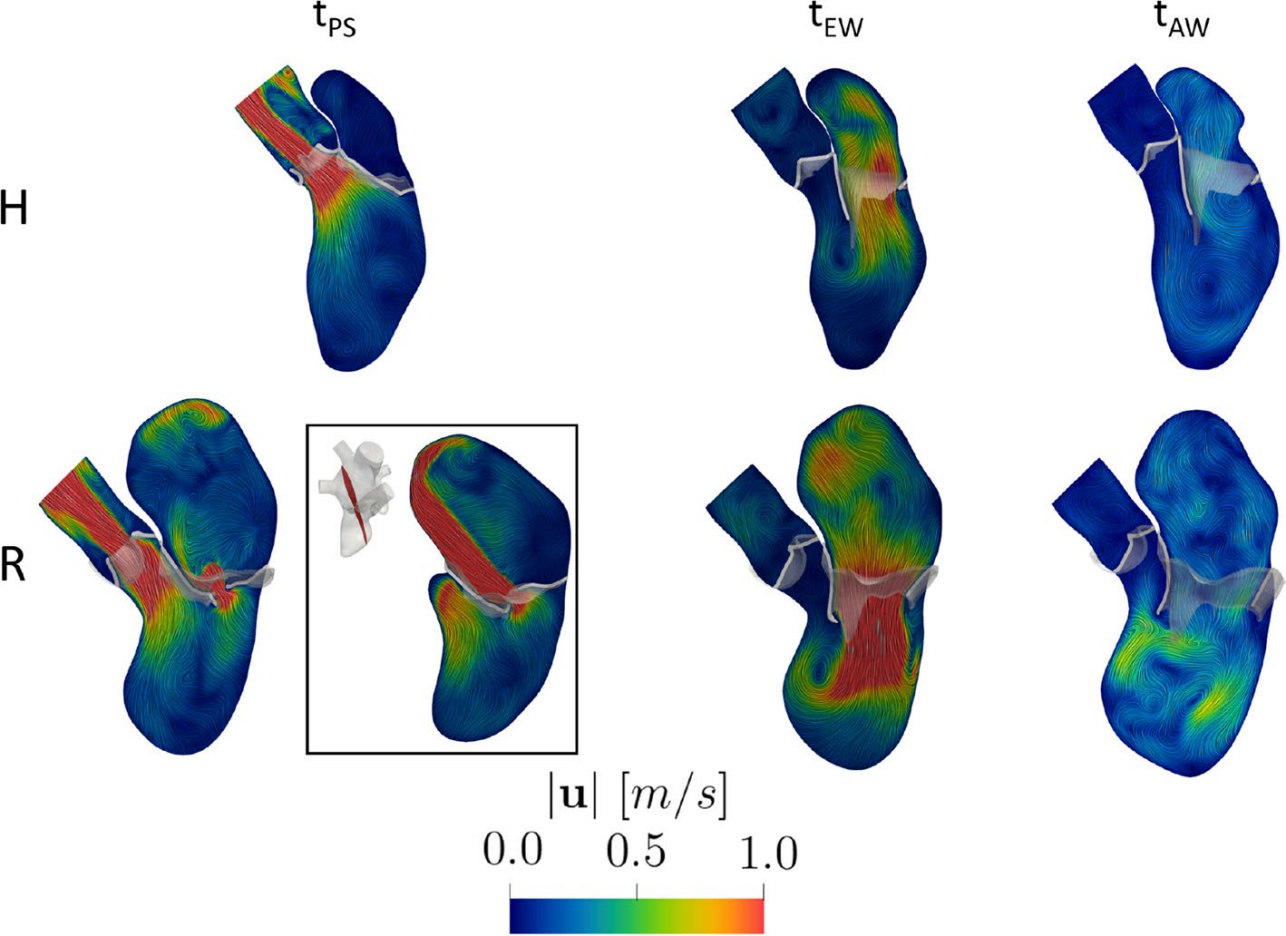
Magnitude of the ensemble velocity at three representative time instants (peak systole $t_{PS}$, peak E-wave $t_{EW}$, peak A-wave $t_{AW}$) over a slice along the 3CH axis in the two scenarios. At $t_{PS}$, we also reported a slice along the 2CH axis (black box) for the regurgitant case

In Fig. 4, we reported the magnitude of the ensemble velocity field on a slice along the 3CH axis at three representative time instants: the instant of peak systole (i.e., maximum AV flow rate) $t_{PS}$, of peak E-wave $t_{EW}$ and of peak A-wave $t_{AW}$. In particular, $t_{PS}=0.15\ s$ and $0.21\ s$, $t_{EW}= 0.45\ s$ and $0.47\ s$, $t_{AW}= 0.80\ s$ and $0.70\ s$, for H and R, respectively.

At $t_{PS}$, we displayed for R also a slice along the 2CH axis (see the black box), to better highlight that part of the ventricle flow went in the atrium resulting in the formation of a regurgitant jet developing along the anterior leaflet and along the LA walls, with a velocity peak of $5.5\ m/s$ at the level of the MV orifice. The maximum velocity through the AV plane was equal to $2.1\ m/s$ for H and $1.5\ m/s$ for R. As expected, the peak of AV velocity was higher in the healthy scenario (Caballero et al. 2018; Obermeier et al. 2022). Notice also the different velocity distributions in the atrium in the two scenarios: In H, no specific velocity patterns were observed, whereas in R the regurgitation promoted chaotic and irregular structure formations. At $t_{EW}$, when the blood flow went from LA to LV, we obtained maximum velocity values through MV equal to $1.08\ m/s$ and $1.73\ m/s$ for H and R, respectively, highlighting the more elevated velocity in R, due to the higher MV flow rate, see Fig. 3B. Furthermore, in both the scenarios we observed the formation of a ventricular vortex ring developing below the anterior leaflet. At $t_{AW}$, the second injection of fluid in the ventricle occurred. The velocity through MV were lower with respect to the ones observed at $t_{EW}$ for both the scenarios. Furthermore, we noticed swirling structures in the ventricle, especially in correspondence with the middle-apex areas, due to vortices formed during the diastasis.

During the E-wave, we calculated the value of EPI in the ventricle, which was equal to 1 and 2 for H and R, respectively, highlighting the better ability of R to washout ventricular blood than H.

**Fig. 5:**
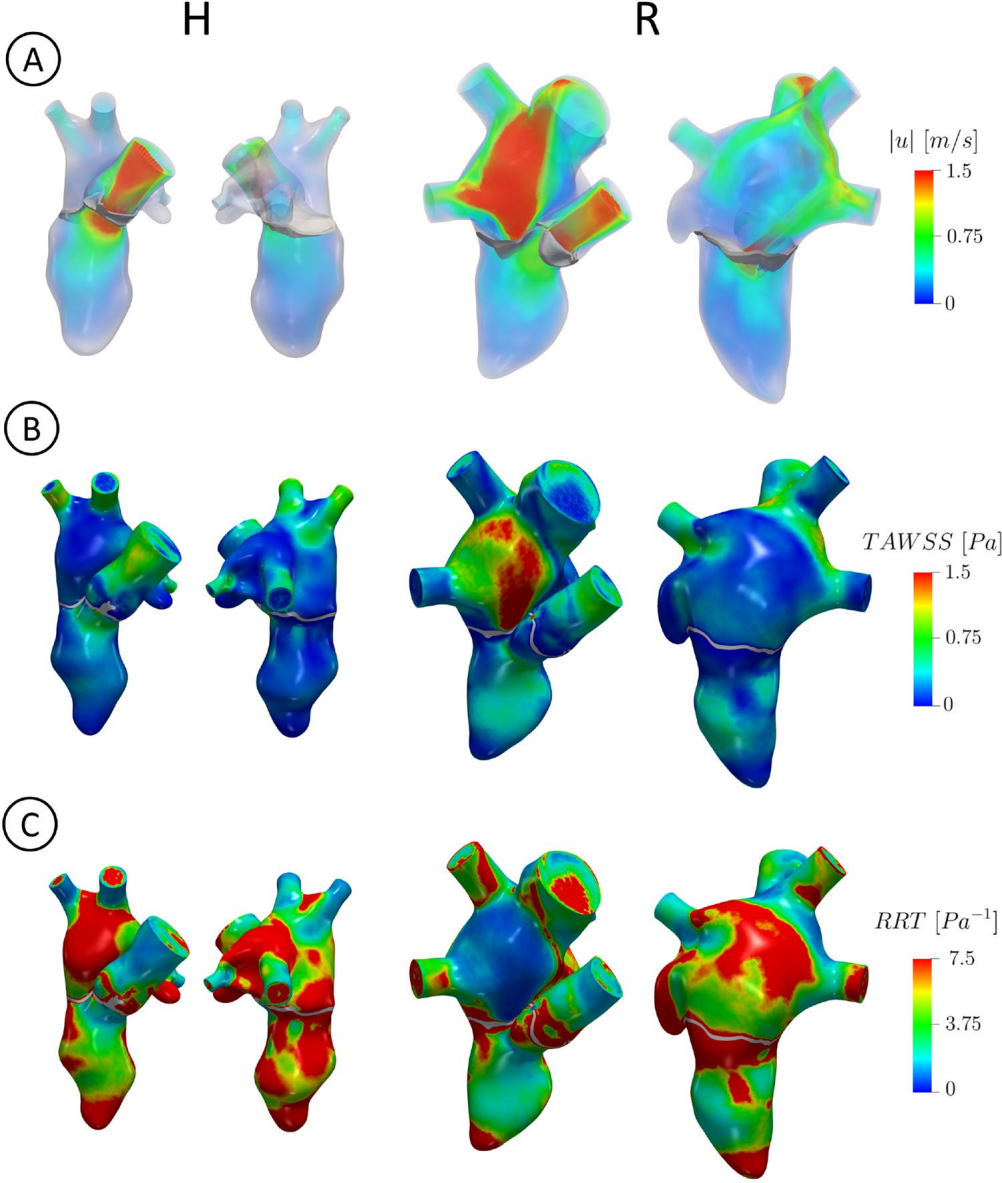
For each scenario in two different views: A: volume rendering of the ensemble velocity magnitude at $t_{PS}$; B: spatial distribution of TAWSS, reported in the end systolic configuration; C: spatial distribution of RRT, reported in the end systolic configuration

In Fig. 5, we reported the volume rendering of the ensemble velocity magnitude at $t_{PS}$ (panel A), the spatial distribution of TAWSS (panel B) and RRT (panel C) for the two scenarios, in two different views.

From this figure, we observed that for R the regurgitant jet gave rise to high velocities, elevated values of TAWSS and low values of RRT where the blood flow scratched and impinged against the atrial walls. Notice also that in the ventricle, TAWSS was slightly higher in R due to larger velocities occurred during the diastolic phase, see Fig. 4. To quantify these differences, we reported in Table 2 the percentage of area with RRT greater than $5\ Pa^{-1}$, computed over the total LV surface, the LV apex, the total LA surface and left atrial appendage (LAA), in the two scenarios. Notice that such values were in any case larger for H than R.

**Table 2:** Values of the quantities of interest computed for each scenario. EPI: E-wave propagation index; Percentage of area with RRT greater than $5\ Pa^{-1}$ evaluated in four different locations (LV, $LV_{apex}$, LA and LAA); $\overline{GTKE}$: average in time of GTKE evaluated in LV and LA; total exposure time of the blood to values of $\tau _{max}>$ $800\ Pa$

		*AREA* ${RRT}>5\ Pa^{-1}\ [\%]$				$\overline{GTKE}\ [mJ]$		duration [ms]
Scenario	$EPI\ [-]$	LV	LV$_{apex}$	LA	LAA	LV	LA	${\tau _{max}>800\ Pa}$
H	1	68	83	48	100	0.20	0.12	0
R	2	40	53	33	100	4.80	7.30	220

We pointed out that the threshold was chosen as representative value to discriminate high and low values of RRT. However, the analysis performed with other thresholds led to the same conclusions (percentage of area above the threshold larger in H).

**Fig. 6:**
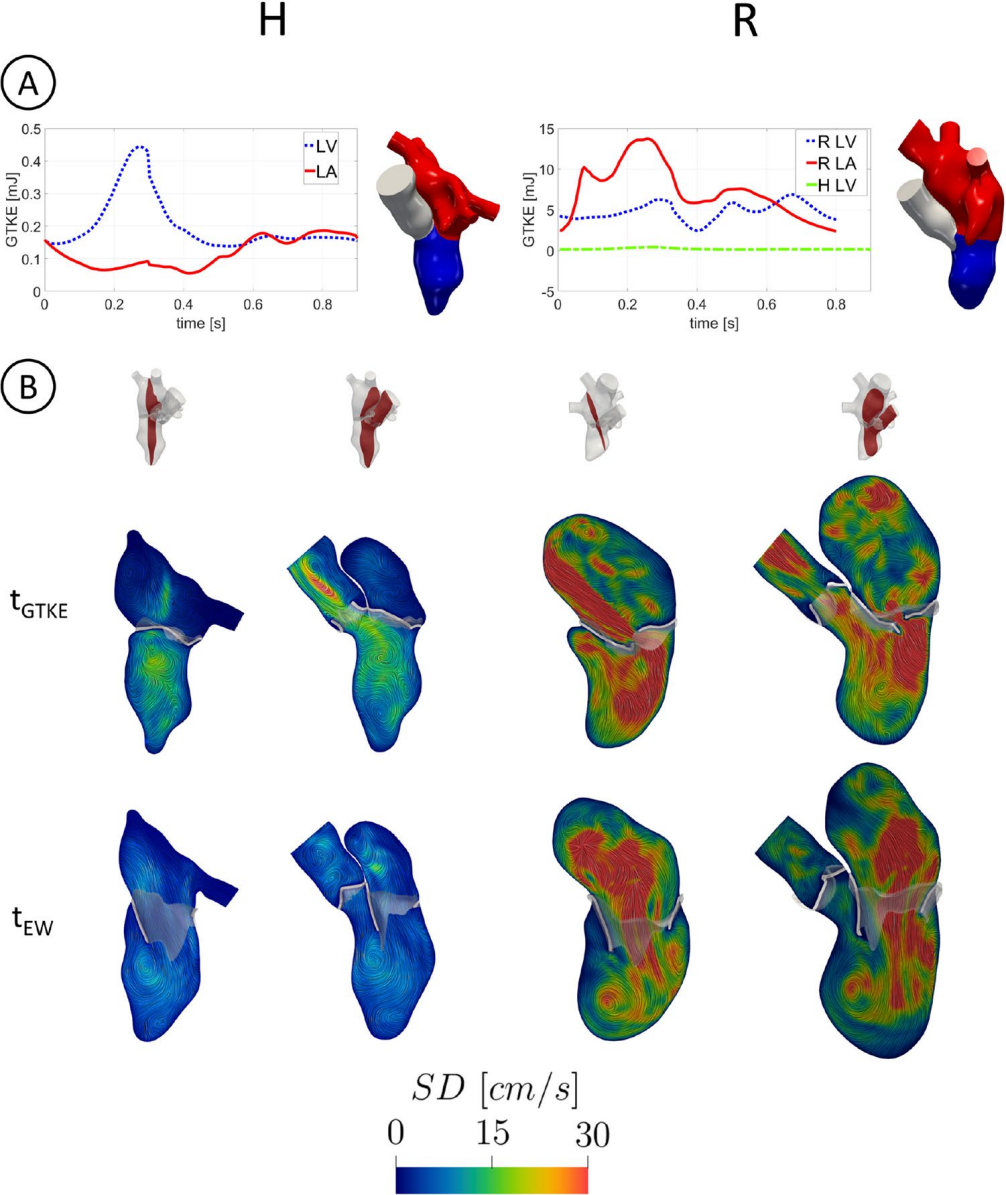
A: trend in time of GTKE evaluated in LV and LA. Notice the different scale used for H (left) and R (right). In the right figure, also the H LV case has been reported for a direct comparison; B: slices along the 2CH (left) and 3CH (right) axis with the velocity SD at $t_{GTKE}$ and $t_{EW}$

In Fig. 6A, we reported, for each scenario, the evolution in time of GTKE evaluated in LV and LA.

We observed that in R GTKE was much larger than in H in all the two chambers, with maximum values in LA at the end of systole due to the regurgitant jet and almost constant values in LV. With $t_{GTKE}$, we referred to the time instant of maximum GTKE, equal to $0.29\ s$ (i.e., at $32\%$ of the heartbeat) and to $0.25\ s$ (i.e., at $31\%$ of the heartbeat) for H and R, respectively. In Table 2, we reported the average in time of GTKE, for both the scenarios confirming larger values for R. In Fig. 6B, we displayed at $t_{GTKE}$ and $t_{EW}$ two slices along the 2CH (left) and 3CH (right) axes with the velocity SD. R featured in any case larger values of SD in all the left heart than H, confirming the greater predisposition of R to develop transition to turbulence. SD values in R at $t_{GTKE}$ were comparable with the ensemble velocity ones, with a peak of $183\ cm/s$ in correspondence with the regurgitant jet and of $39\ cm/s$ in LV. Instead, in H the maximum SD value was equal to $17\ cm/s$ in LA and to $19\ cm/s$ in LV. Notice also a peak of $31\ cm/s$ located in AR. At $t_{EW}$, the fluctuations were mainly present in the center of LA with a peak of $55\ cm/s$ in R. Instead in H, we noticed very low SD values with a peak in the LA center of $13\ cm/s$.

**Fig. 7:**
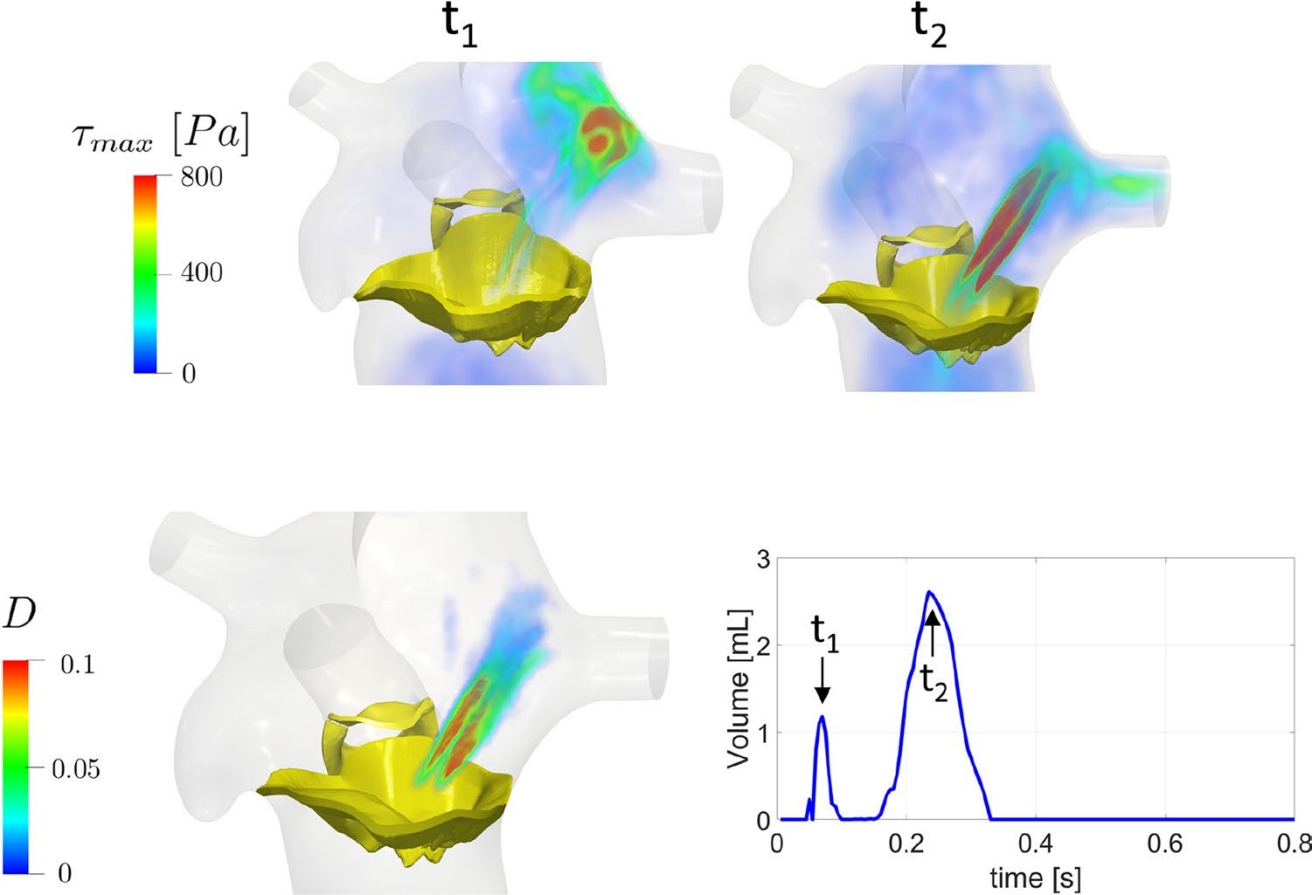
Top: volume rendering at two different time instants $t_1$ and $t_2$ of the maximum tangential stress $\tau _{max}$ quantifying the possible damage caused to blood cells in the regurgitant scenario; Bottom, left: Percentage $D(\textbf{x})$ of the cardiac cycle duration during which $\tau _{max} > 800\ Pa$; Bottom, right: time evolution of the volume of blood characterized by $\tau _{max} > 800\ Pa$ and identification of $t_1$ and $t_2$ as the peak instants

In Fig. 7, top, we reported for the regurgitant scenario the volume rendering of the fluctuating forces $\tau _{max}$ at the instants where the volume of blood characterized by $\tau _{max}> 800\ Pa$ featured its peaks, $t_1 = 0.07\ s$ and $t_2 = 0.24\ s$, see Fig. 7, bottom, right. In the latter figure, we reported for each time the amount of volume where blood is exposed to values of $\tau _{max}$ greater than $800\ Pa$. We observed values of $\tau _{max}$ greater than $800\ Pa$ when the regurgitant jet impinged against the atrial walls ($t_1$) and when a rapid deceleration of the regurgitant flow through MV occurred ($t_2$), see also Fig. 3B. In Fig. 7, bottom, left, we reported the percentage *D* of the cardiac cycle during which $\tau _{max} > 800\ Pa$: $D(\textbf{x}) = \overline{t}/T$, where $\overline{t}$ is the amount of time along the cardiac cycle during which $\tau _{max} > 800\ Pa$ in point $\textbf{x}$. These results highlighted that the region most exposed to $\tau _{max} > 800\ Pa$ is in correspondence with the regurgitant jet and that its exposure time is significant (more than $15\%$ of the heartbeat duration). From Fig. 7, bottom, right, we observed that during the diastolic phase the volume with elevated $\tau _{max}$ was always null. We also computed the total exposure time to values greater than $800\ Pa$, which was equal to $220\ ms$ for R and $0\ ms$ for H, suggesting the absence of hemolysis risk in the healthy scenario (see Table 2).

To assess the accuracy of the results obtained for subject H, we compared the values of the ensemble velocity computed by the numerical simulation with the available ECD measures in the locations P1, P2 and P3 reported in Fig. 2A. In Table 3, we reported a comparison of the peak values in the three locations.

**Table 3:** Comparison for the healthy case between the measurement of ECD and the values of the numerical simulation (SIM) in the three points of Fig. 2A. $\Delta$ is the relative discrepancy

	P1			P2			P3		
Scenario	$ECD\ [m/s]$	$SIM\ [m/s]$	$\Delta \ [\%]$	$ECD\ [m/s]$	$SIM\ [m/s]$	$\Delta \ [\%]$	$ECD\ [m/s]$	$SIM\ [m/s]$	$\Delta \ [\%]$
H	1.25	1.20	4.0	0.85	0.80	5.9	0.62	0.60	3.2

The maximum relative error $\Delta$ was found in P2 (5.9%), whereas in P1 and P3 the error was no larger than 4.0%.

**Fig. 8:**
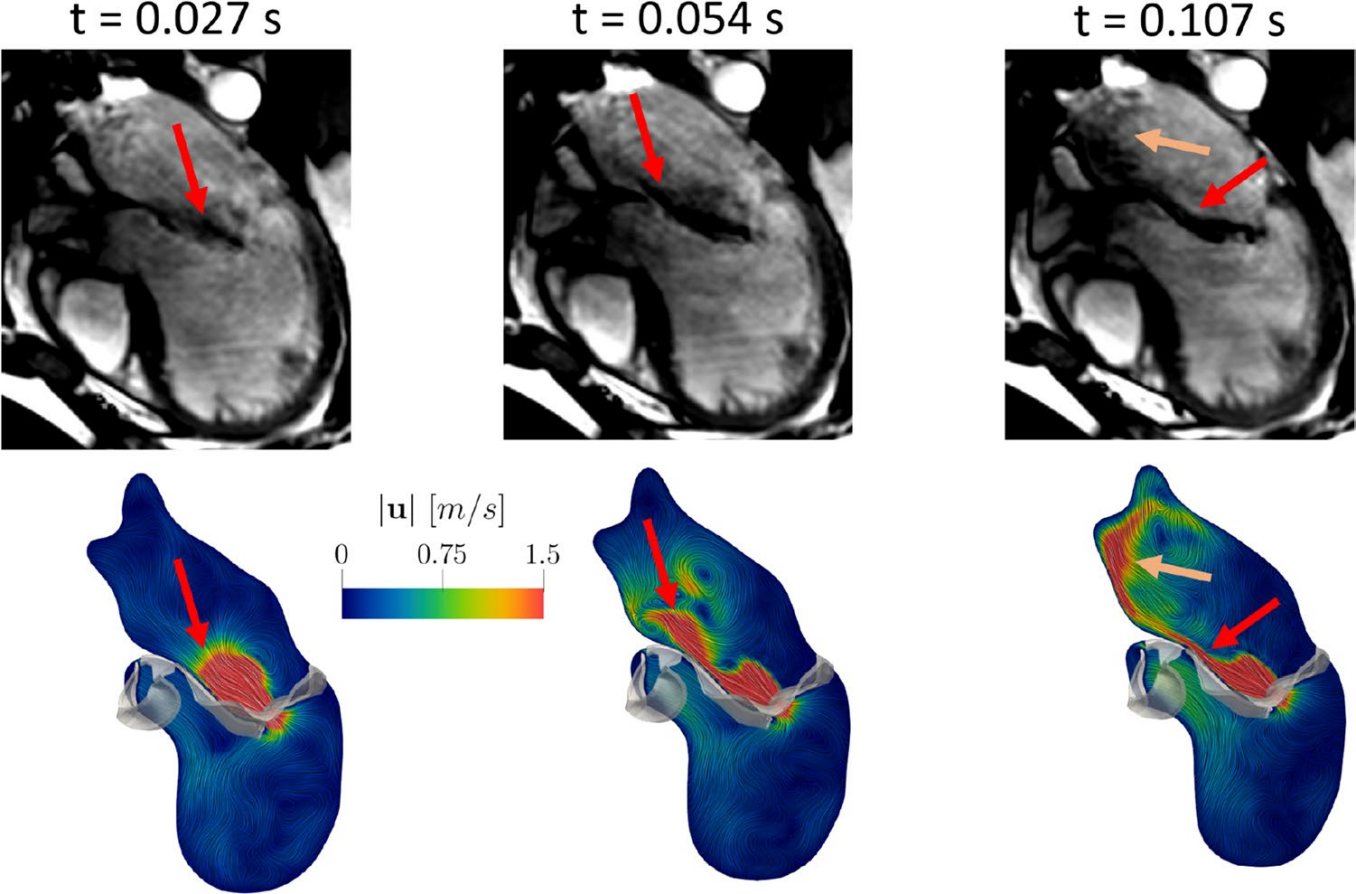
Comparison of the ensemble velocity pattern with Cine-MRI at three representative frames in the regurgitant scenario. The arrows highlight the good agreement between computations and measures

In Fig. 8, we reported a qualitative comparison of the regurgitant flow pattern obtained by the numerical simulation (bottom) with cine-MRI (top) at three representative frames in R. In particular, in the cine-MRI views the regurgitant jet was detected by the areas characterized by a darker color, representing high blood flow velocities.

Notice the good qualitative agreement between computations and images, highlighting that the regurgitant jet firstly developed along the anterior leaflet (Frames 1 and 2) and then along the atrial walls assuming also a swirling structure (Frame 3).

## Discussion

In this work, we performed an image-based computational fluid dynamic study in the left heart of a healthy subject and of a patient with a severe mitral regurgitation (MVR), with the aim of comparing the transition to turbulence, the risk of hemolysis and the risk of thrombi formation. The motion of the complete left heart (LH) (i.e., left ventricle (LV), left atrium (LA) and aortic root (AR) internal wall surfaces, together with aortic valve (AV) and mitral valve (MV)) during the whole heartbeat was reconstructed and prescribed to DIB-CFD simulations from multi-series cine-MRI.

At the best of our knowledge, this is the first work using the patient-specific geometry motion of LV, LA, AR in a mitral regurgitant DIB-CFD simulation. Moreover, ours is the first work which included also MV and AV geometries in a (general) DIB-CFD simulation. In particular, for the healthy case the majority of the previous DIB-CFD studies did not include LA or AR or employed template geometries (Obermeier et al. 2022; Fumagalli et al. 2020, 2022; Seo et al. 2014; Su et al. 2016; Bennati et al. 2023). In Chnafa et al. (2014, 2016); Karabelas et al. (2022) the authors reconstructed all the patient-specific LH walls of a healthy subject, where, however, MV and AV in Karabelas et al. (2022) and AV in Chnafa et al. (2014, 2016) were geometrically modeled as planes in their FC configuration and disappeared in the FO configuration. For the regurgitant case, no patient-specific LA and AR motions together with the geometries of AV and MV could be found in previous studies.

In our work, the entire reconstruction of the left heart internal wall surfaces and valves was performed by combining different (multi-series) cine-MRI at disposal: the LV Long Axis, the LV Short Axis, the AR Short Axis (when available) and the MV Rotated series. The latter stand out from the other series because they are based on a *radial sampling* (while the other series on a *Cartesian sampling*) which consist in 2D Long Axis series of 18 evenly rotated planes (one every 10 degree) around the axis passing through the MV center and aligned with the LV apex, proposed for the first time in Stevanella et al. (2011) for a pure structural analysis. Such images represented advanced acquisitions not acquired in the clinical routine, see Fig. 1, which allowed us to obtain detailed 3D geometry motion of LA, AR, MV and AV, in addition to ventricle motion for which the LV Short and Long Axis series are sufficient.

Owing to this specific and detailed imaging, we were able to quantitatively describe physiological and pathological/MVR mechanisms such as prevention from thrombi, transition to turbulence and hemolysis formation. In particular, for the first time at the best of our knowledge, we investigated by means of a computational study how the regurgitant jet could promote the washing out of regions with stagnant flow in LA, reducing the risk of thrombosis with respect to a healthy scenario. Our results highlighted that the regurgitant jet scratched against the atrial walls, see Fig. 5A, resulting in more elevated values of TAWSS wrt H, see Fig. 5B. This led to lower values of RRT in the atrium (Fig. 5C) indicating that there is little stagnation in those regions and thus promoting the washing out of blood. This was also confirmed by the values reported in Table 2. Such results are in line with the literature according to which MVR is responsible for reducing possible risks of thrombi formation in LA wrt to an healthy case (Fukuda et al. 2011; Cresti et al. 2019).

Notice, however, that RRT in the left atrial appendage was elevated in both the subjects, confirming that, even in the presence of MVR, LAA could be one of most common site for cardiac thrombus formation (Yaghi et al. 2015). Moreover, we investigated the mechanism of washing out occurring in the ventricle during the E-wave. In particular, in the regurgitant scenario there was a significant LV apical washout due to high blood flow coming from MV, see Figs. 3B and 4 as confirmed by the value of EPI and of low RRT areas, see Table 2. In particular, the higher velocities through MV in R, see Fig. 4, allowed the blood to reach the ventricle apex rapidly and with high energy before diastasis, allowing to remove possible areas of stagnant flow. These results about the comparison of EPI, RRT and velocity through MV between R and H are consistent with clinical observations highlighting that MVR is in general associated with an increased diastolic flow rate through MV (wrt a physiological scenario) due to LV and LA dilation (Delahaye et al. 1991; Thomas et al. 1998; Harfi et al. 2017). Thus, patient R promoted a more relevant washout, with respect to subject H, of the blood flow in LA during the systolic phase and in LV during the E-wave, leading to regions more protected from the possible formation of clots. Our conclusions are in agreement with clinical studies (Kalaria et al. 1998; Cresti et al. 2019; Van Laer et al. 2021).

Second, we investigated the transition to turbulence in the MVR case. So far, at the best of our knowledge, the turbulent effects were investigated only in physiological left hearts (Chnafa et al. 2014, 2016). By employing a LES model, we showed that the regurgitant scenario featured high velocity fluctuations among the heartbeats, resulting in elevated velocity standard deviation, see Fig. 6 and the values of GTKE reported in Table 2. In particular, the maximum fluctuations in LA occurred for R at late systole, as also reported in Dyverfeldt et al. (2011), with a GTKE peak value equal to $14\ mJ$, which falls in the range $(13,37)\ mJ$ found in Dyverfeldt et al. (2011). Moreover, the ratio of the average GTKE in LA between R and H was equal to 61, see Table 2, confirming that the presence of the regurgitant jet promoted more turbulence in R. We noticed also elevated transition to turbulence for R in LV, especially during the rapid deceleration of the systolic blood flow (Fig. 3B), during the E-wave due to the formation of a ventricular vortex ring and to the impingement of the diastolic jet against the LV apex (Fig. 4), and during the A-Wave due to the mixing of blood after diastasis (Fig. 4). The ratio of the average GTKE in LV between R and H was equal to 24, highlighting how the regurgitation promoted a great amount of turbulence also in LV. We point out that, in our results, the transition to turbulence in LV was more pronounced for R because of the larger heart dimension resulting in higher ventricular blood flow. Since the BSA of the two subjects was very similar (see Table 1), we argue that the larger LH dimensions of patient R were due uniquely to MVR, which is known to be correlated with progressive remodeling and dilation (Delahaye et al. 1991). In this respect, we noticed that the ratio between R and H LV diameters at the end diastolic configuration was equal to 1.25, a value in agreement with medical studies reporting that severe MVR dilated the left ventricle diameter by a factor of 25-30% (Tribouilloy et al. 2009; Narayanan et al. 2014).

Third, we observed that the definition of $\tau _{max}$ (see Sect. 2.5) implies that large transition to turbulence (and thus large values of $\tau _{max}$) promotes hemolysis risk for the MVR case . In particular, we found high values of $\tau _{max}$ (greater than $800\ Pa$, identified as a risk threshold Lu et al. 2001) and of time exposure to elevated $\tau _{max}$ in correspondence with the regurgitant jet, see Fig. 7, top and bottom, left. We highlighted two different mechanisms (Fig. 7, bottom, right) that provoke large $\tau _{max}$: at $t_1$ the fragmentation of the regurgitant jet against the atrial walls; and at $t_2$ the rapid deceleration of the blood flow through MV, see also Fig. 3B. These two different mechanisms were also described by clinical studies (Yeo et al. 1998; Sugiura et al. 2018). Furthermore, according to Lu et al. (2001), regions exposed to value of $\tau _{max}$ larger than $800\ Pa$ for more than $1\ ms$ could experience the conditions of promoting hemolysis. In our R case, we found a value of $220\ ms$ (i.e., 70% of the systolic phase), see Fig. 7, bottom, right and Table 2. Thus, our results are in accordance with clinical studies highlighting that the regurgitant jet may create the conditions to promote hemolysis, especially in case of severe MVR (Sugiura et al. 2018).

The blood velocity results found in this work for the healthy case have been validated against ECD measures acquired in the subject in three different locations. In particular, although far to establish a complete validation, they are very promising since they highlighted a good accuracy (with a maximum error of 6%, see Table 3) between the peak values. Furthermore, we reported a qualitative comparison of the regurgitant flow pattern obtained by the numerical simulation with the cine-MRI, see Fig. 8. We noticed a good agreement between the cine-MRI and the numerical simulations. In particular, the direction of the regurgitant jet in the numerical simulation was also in accordance with the Carpentier’s functional classification for which, in case of MVR due to a prolapse, the jet is directed away from the pathological leaflet, in our case the posterior one (Carpentier 1983; Stewart et al. 1992).

To support the other results and the physiopathological implications, we compared our findings with the literature. In particular, in the healthy scenario the value of the maximum pressure drop $\Delta P_{MV}= 3.6\ mmHg$ computed across MV falls down in the range $\simeq (2.0,4.0)\ mmHg$ found in Firstenberg et al. (2000). In the regurgitant scenario, we compared our velocity findings with measures in the case of a severe MVR. We found a value of the maximum velocity through MV at systole equal to $5.52\ m/s$, in accordance with the range $(5.00,6.00)\ m/s$ (Dyverfeldt et al. 2011) and during the E-Wave equal to $1.73\ m/s$, in accordance with the range $(1.18,1.77)\ m/s$ (Thomas et al. 1998). Notice that the E-Wave peak velocity value is used in clinics to assess the severity of MVR. Notice also that the MV flow reversals detected at diastasis ($t\simeq 0.59\ s$) and before the end of the heartbeat ($t\simeq 0.77\ s$, see Fig. 3B) were also found in other computational studies (Broomé et al. 2013; Caballero et al. 2018; Karabelas et al. 2022).

## Limitations

Some limitations characterized this work:

1. We considered only two subjects. This was a consequence of the fact that we used advanced (not daily available) images series elaborated in order to perform highly accurate DIB-CFD simulations. This allowed us to extract interesting general physiopathological findings of the healthy and regurgitant scenarios. As a consequence, our reconstruction technique is not suitable to study a wide range of specific subjects, for which standard images are enough;
2. We did not model the valve dynamics; instead, we considered an instantaneous valve opening and closure. However, the opening and closing valve time duration is less than 5% of the heartbeat (Yoganathan et al. 2004). Thus, the opening and closure of the valves can be considered as instantaneous as a first approximation. The degree to which a patient-specific valve dynamics may affect the velocity field and the transition to turbulence is currently under study;
3. We did not include the subvalvular apparatus (papillary muscles + chordae tendineae) in the LV geometry. This is a common choice, adopted also in Fumagalli et al. (2020); Su et al. (2016); Fumagalli et al. (2022); Bavo et al. (2016, 2016) due to the difficulty to reconstruct the papillary muscles and chordae tendineae from MR images. However, their influence on the quantities of interest, in particular on transition to turbulence and on the ventricular WSS (Sacco et al. 2018), should be relevant and it will be the subject of future studies;
4. We did not considered the MRI beat-to-beat variations in terms of acquired displacements and heart rate. Indeed, the average of MRI acquisitions along more heartbeats should provide more reliable input data for DIB-CFD. However, this may result in prolonged exposure time during the acquisitions of cine-MRI that is not advisable for patients with heart pathologies.
5. Our meshes did not include any boundary layer to better capture the blood dynamic behavior close to the myocardial wall. This should be considered in future studies. However, we notice that our mesh resolution obtained after a refinement study was able to satisfy the Pope criterion.

## Footnotes

1 provided by the Short Axis series of the left ventricle, see below

2 See Fig. 3B in the Results section
